# The Influence of Pro-inflammatory Cytokines and Genetic Variants in the Development of Fibromyalgia: A Traditional Review

**DOI:** 10.7759/cureus.10276

**Published:** 2020-09-06

**Authors:** Mercedes Maria Peck, Ruchira Maram, Alaa Mohamed, Diego Ochoa Crespo, Gurleen Kaur, Ibtisam Ashraf, Bilal Haider Malik

**Affiliations:** 1 Internal Medicine, California Institute of Behavioral Neurosciences & Psychology, Fairfield, USA; 2 Internal Medicine, Arogyasri Healthcare Trust, Hyderabad, IND; 3 Internal Medicine, Memorial Hermann Medical Center, Houston, USA; 4 Internal Medicine, Clinica San Martin, Azogues, ECU; 5 Neurology, California Institute of Behavioral Neurosciences & Psychology, Fairfield, USA; 6 Internal Medicine, Shalamar Institute of Health Sciences, Lahore, PAK

**Keywords:** fibromyalgia, pro-inflammatory cytokines, cytokines, fibromyalgia and inflammation, fibromyalgia and cytokines, interleukin 8, interleukin 17, pain and genetics, genetic and sleep disturbances, genetics and insomnia

## Abstract

Fibromyalgia is a complex syndrome characterized by widespread chronic pain, without any obvious etiology, and it is often accompanied by a constellation of symptoms such as fatigue, sleep disturbances and cognitive dysfunction, to name a few. The syndrome may be associated with a variety of autoimmune and psychiatric conditions. Fibromyalgia can occur with other musculoskeletal pathologies and its symptoms can overlap with other chronic painful conditions such as chronic myofascial pain syndromes seen in cervical and lumbar spinal osteoarthritis and degenerative disc disease. Gene polymorphisms have been related to a decreased pain threshold and an increased susceptibility to disorders associated with chronic pain. Some of those genetic variants might trigger the onset of fibromyalgia. Researchers are looking into the possible factors that might contribute to its pathophysiology. It is important to study the connections between pro-inflammatory cytokines and genetic variants in pain-related genes and their roles in predisposition and development of fibromyalgia. The objective of this review article is to provide a brief overview of the pro-inflammatory cytokines commonly associated with fibromyalgia, as well as to look into the genes that have shown some level of involvement in the development of fibromyalgia and its symptomatology.

## Introduction and background

The prevalence of fibromyalgia is around 0.8% to 5% worldwide. In the United States it ranges from 2% to 8%, affecting approximately 5-10 million of the adult population; females represent the majority of the reported cases [1-3]. To date, there is no detailed documentation about significant ethnic variations in fibromyalgia prevalence [2]. Even though fibromyalgia is a common persistent pain disorder, it still represents a diagnostic challenge to many physicians not only due to lack of a "gold standard' method to help make an accurate diagnosis but also due to its complexity and inexplicable etiology [1-2]. Because of its tangled nature, it might take up to two or more years to make a precise fibromyalgia diagnosis. Affected patients see approximately an average of 3.7 healthcare providers during that time [2].

Fibromyalgia is defined as widespread pain for more than three months accompanied with multiple symptoms such as fatigue, cognitive dysfunction, unrefreshing sleep, and somatic symptoms. Other manifestations that can be present are dry mouth, dry eyes, musculoskeletal stiffness and tenderness to touch [2-4]. Fibromyalgia is associated with multiple comorbidities, particularly with psychiatric and rheumatology disorders [1-2]. This condition is still under-diagnosed and under-treated, which might be in part due to the overlapping symptoms of other diseases and disorders. These disorders can mimic the symptomatology observed in fibromyalgia, making it hard to give a timely precise diagnosis [2,4].

The 2010 American College of Rheumatology Fibromyalgia criteria was used to approach a diagnosis of fibromyalgia, which comprises two elements, the 0-19 widespread pain index (WPI) and the 0-12 symptom severity (SS), which can be joined to form a 0-31 index. One of its problems was that it did not take into account the spatial distribution of painful sites in the body [2,5]. The authors of the revised 2016 criteria resolved this problem by requiring patients to bear pain in four out of five regions, called "generalized pain”. This is currently the most appropriate diagnostic tool [2].

The pathogenesis of fibromyalgia is still poorly understood [6]. Several factors are being considered to play an essential role in the pathophysiology of this condition [7]. Some hypotheses on central and peripheral nervous system dysregulation and the role of pro-inflammatory cytokines such as interleukins (ILs) and their assistance with nociception have been proposed [7-8]. Genetic variants in pain-related genes have also been linked to fibromyalgia susceptibility [7]. The treatment for fibromyalgia is focused on pharmacological and behavioral interventions to reduce its symptomatology [9].

The purpose of this traditional review article is to provide a comprehensive overview of the pathophysiology of fibromyalgia, as well as to give insight into the role played by pro-inflammatory cytokines released by the immune system in the pathogenesis of fibromyalgia. Also, it seeks to describe the implication of genetic variants associated with an increased risk of developing fibromyalgia.

## Review

Fibromyalgia overview

One hypothesis suggested that in fibromyalgia patients, inflammatory cytokines could drive disturbances in neural networks during the interaction of the nervous system with immune cells, which eventually could lead to increased central and peripheral sensitization as well as neuroinflammation [10-12]. The epidemiological profile of fibromyalgia shares some similarities observed in various autoimmune disorders, such as its peak of incidence amongst middle-aged females, “with a female to male ratio oscillating from 1.5:1 to 10:1” [3,13]. Also, it is not unusual to see concomitant autoimmune comorbidities in people diagnosed with fibromyalgia, thereby adding some support to this hypothesis [3]. Several research results about the role and levels of pro-inflammatory cytokines in fibromyalgia have shown controversial results. Some have demonstrated a positive association between serum and cerebrospinal fluid levels of cytokines and pain severity while others have shown little to no evidence in the correlation between them [11,14-18]. Throughout the years it has been well documented that inflammatory mediators such as cytokines released by immune cells play an essential role in the generation of pain. Similarly, the implication of such mediators in multiple conditions associated with chronic pain and inflammation has been evidenced [18]. Despite being “classified as a rheumatic disease, fibromyalgia is mostly treated as a neurological problem” and not as a rheumatic problem [3]. The immune system is just one of several factors that might be involved in the pathophysiology of fibromyalgia. Due to the strong association of genetic variants with chronic pain disorders, pain-related gene polymorphisms are now considered to be key in the mechanism behind the pathogenesis of fibromyalgia [2]. Single nucleotide polymorphisms identified in genes coding for neurotransmitters (serotonin, dopamine, etc.) could contribute to the dysfunction of nociceptive pathways observed in patients with fibromyalgia. These polymorphisms are also crucial in the development of many symptoms seen in those patients such as depression and anxiety [7].

Cytokines

Cytokines are molecules released by cells that are a part of the immune system and non-neuronal cells such as epithelial cells [12]. These molecules are the mediators responsible for the inflammatory reactions observed in both the innate and adaptive immune responses. Additionally, they can stimulate nociceptor neurons, therefore sensitizing pain network pathways [12,19]. Cytokines can be classified as pro-inflammatory (e.g. IL-17), which are involved in painful, acute, and chronic inflammatory conditions or as anti-inflammatory mediators providing an analgesic effect (example IL-4, IL-5). Some cytokines can have both pro- and anti-inflammatory actions. The type of action will depend on what kind of receptor is being activated (e.g. IL-6) [16-17,19]. The widespread pain and hypersensitivity seen in patients with fibromyalgia could be due to an imbalance between pro-inflammatory and anti-inflammatory cytokines, which could provoke the induction and maintenance of pain [14,17]. The following pro-inflammatory cytokines could be essential in the pathophysiology of fibromyalgia and many studies have been done in order to understand their possible involvement in fibromyalgia. We will briefly discuss their biological action.

Interleukin-1 beta (IL-1β)

Macrophages and monocytes present in the periphery and microglia cells in the central nervous system are responsible for the synthesis and release of IL-1β [20]. Inflammasomes are complexes of proteins that control the synthesis of IL-1β. When they sense noxious stimuli they induce a cascade of reactions leading to the activation and subsequent release of IL-1β. Eleven isoforms integrate the IL-1 family and all of them have the ability to induce inflammation [21]. IL-1β is characterized by its powerful pro-inflammatory effect in multiple tissues. Its signal on nociceptors leads to an improvement in the conduction and transduction of pain through various ion channels [12,20-21]. An augmented activity of IL-1β is involved in many autoimmune disorders featuring pain such as inflammatory bowel disease, gout, multiple sclerosis, and rheumatoid arthritis [12,21].

IL-1β's association with fibromyalgia pathophysiology has been the subject of both human and animal studies. The results of several studies have shown the presence of increased levels of IL-1β in patients diagnosed with fibromyalgia compared to controls, giving some support to this linkage [17,22]. One study demonstrated that using a low dose of naltrexone (an atypical anti-inflammatory drug) for eight weeks diminished the overall symptomatology of fibromyalgia by 18% and also showed a 15% reduction of related pain. This study found that naltrexone suppressed the levels of various cytokines (including IL-1) known for their contribution to nociception, allodynia, and hyperalgesia. However, this study has some limitations, including its short duration, small sample size, and the lack of a control group, which cause some uncertainty over its general application [23]. Despite multiple favorable findings about the possible involvement of IL-1β in fibromyalgia, the results of some studies have not shown support of the bond between the levels of IL-1β and fibromyalgia [14,17].

Interleukin-6 (IL-6)

Macrophages, monocytes and dendritic cells produce and release IL-6 during the innate immune response. Also, mast cells can release IL-6 without the need for degranulation [11-12,24]. IL-6 has various key functions in the adaptive immune response, one of which is the induction of B lymphocytes to produce immunoglobulins. IL-6 is pleiotropic inflammatory cytokine with a wide range of biological functions. When it binds to its IL-6 receptor (IL-6R) it forms a complex which then interacts with a membrane signal transducer protein, glycoprotein 130 (gp130), promoting the initiation of multiple intracellular signaling pathways such as the Janus Kinase signal transducer and activator of transcription 3 (JAK-STAT3) pathway. This drives the expression of the gene coding for acute-phase proteins [24-25].

IL-6 contributes to the generation of pain by central sensitization and nociceptive neurons sensitization. Moreover, it has been tightly linked to nociceptive plasticity through the improvement of translation in sensory neurons [12,25]. IL-6 is a cytokine highly expressed in the central nervous system, notably in the hypothalamus [24]. Previous studies on pathological pain found high expression levels of gp130, IL-6 and IL-6R in the dorsal root ganglion and in the spinal cord [12,15,25]. Elevated synthesis of IL-6 and dysregulation in the IL-6 receptor signaling are associated with disease pathology, especially those manifested with pathological pain [24-25]. Like many other cytokines, IL-6 is thought to be an essential part in the pathophysiology of fibromyalgia. However, the precise mechanism and role of this cytokine in widespread pain associated with fibromyalgia are still a subject of study [14].

Interleukin-17 (IL-17)

The IL-17 family is a group of six structurally related cytokines (IL-17A-F) [26]. IL-17A, also called IL-17, besides being the prototype of the IL-17 family, is the most understood and studied member. It can be secreted by several immune cells, T helper lymphocytes (Th17 cells) for example [26-28]. IL-17 has a critical role in the inflammatory response. It promotes the production of chemokine and granulocyte colony-stimulating factors and helps in the recruitment of immune cells on-site of infections [26,28]. It is well established that IL-17 dysregulation contributes to the pathogenesis of many autoimmune disorders such as multiple sclerosis and can also lead to several chronic inflammatory conditions [12,26-28]. Elevated plasma levels of IL-17 as well as tumor necrosis factor (TNF) have been observed in patients diagnosed with fibromyalgia [8].

Interleukin-8 (IL-8)

A member of the elastin-like recombinamer C-X-C (ELR CXC) chemokine family, IL-8, also known as C-X-C motif ligand 8 (CXCL8), is synthesized by immune, endothelial, and airway smooth muscle cells. IL-8 is an important cytokine during the inflammatory response [29]. One of its roles is the recruitment and activation of innate immune cells such as neutrophils and granulocyte [29-30]. Equally, it is implicated in the tumorigenesis of several malignancies [29]. As mentioned above IL-8 is crucial in inflammatory processes and also has a vital function in the mediation of pain present in various chronic inflammatory disorders such as rheumatoid arthritis. It makes sense why many studies focus on the involvement of IL-8's biological effects in fibromyalgia [19]. Previous studies in patients with fibromyalgia have shown increased levels of IL-8 in both blood and cerebrospinal fluid (CSF) [8,19,31-32]. Augmented sympathetic system activity has been linked with a marked release of IL-8 and has also been related to pain mechanics. It is valuable to mention that an elevated sympathetic tone seems to be present in fibromyalgia patients. Many symptoms such as fatigue, hyperalgesia, and allodynia observed in several inflammatory conditions are attributed to the effect of pro-inflammatory cytokines, amongst them IL-8. These same symptoms are also seen in patients with fibromyalgia, suggesting that IL-8 might be part of the underlying pathophysiology of fibromyalgia [32].

Tumor Necrosis Factor alpha (TNFα)

The tumor necrosis factor family includes TNFα, TNFβ, and cluster of differentiation 40 ligand (CD40L) [19]. TNFα is a pro-inflammatory mediator produced by various immune cells but mainly secreted by macrophages in the periphery and microglia in the central nervous system, where it can influence neuronal networks in order to module pain signaling [12,19]. TNFα release drives the production of other types of cytokines [19]. TNFα is a critical pro-inflammatory cytokine in both acute and chronic inflammation. Furthermore, it has the potential to modulate pain signaling when TNFα bonds specifically to its TNF receptor 1 (TNFR1) [12,18]. During emotional and physical stress, high levels of substance P and corticotropin-releasing hormone are released. Both substances are tightly implicated in the induction of inflammation and they also have the potential to stimulate mast cells to secrete TNFα. It is worth mentioning that one study found elevated plasma levels of TNFα, substance P and corticotropin-releasing hormone in fibromyalgia patients when compared with controls [11].

Genetics and fibromyalgia

Pain and associated conditions, such as fibromyalgia, frequently aggregate in families [33-36]. It has been well documented in multiple studies that inheritance of pain-related genes contributes up to 50% in the development of chronic pain. To date, hundreds of genes related to pain have been found. Many of these genes are undergoing studies to clarify their role in the generation of pain hypersensitivity and to understand how their effect can affect the expression and function of multiple proteins responsible for regulating pain response (Table 1-3) [7,33,37]. As many other chronic pain diseases, the etiology of fibromyalgia is most likely the outcome of multiple interactions between psychological, neurobiological, environmental triggers and genetic susceptibility, the latter in particular due to the observed, high familial predisposition in fibromyalgia [2,35-36]. First-degree relatives of patients with fibromyalgia have an 8.5-fold increased risk of developing fibromyalgia [35]. Chronic widespread pain along with hypersensitivity to sensory stimuli are the most notable symptoms in fibromyalgia, therefore cardinal gene variants associated to pain are one of the major topics for geneticists looking into the etiology of fibromyalgia. However, besides pain-related genes, there are genes associated with the development of fatigue and sleep disturbances, and also genetic polymorphisms that contribute to more than one symptom [38-39]. The following tables show the most frequent genetic variants associated with chronic pain disorder and possibly with fibromyalgia as well.

**Table 1:** Neurotransmitter Genes Associated to Fibromyalgia 5-HT2A: 5-hydroxytryptamine receptor 2A, 5-HTT: 5-hydroxytryptamine transporter, SLC6A4: solute carrier family 6 member 4, rs: reference single nucleotide polymorphism, 5-HTTLPR: serotonin-transporter-linked polymorphic region, DRD4: dopamine receptor D4, DAT1: dopamine transporter 1, SLC1A5: solute carrier family 1 member 5, SCL25A22: solute carrier family 25 member 22, GABRB3: γ-aminobutyric acid receptor β3 subunit, GABA: γ-aminobutyric acid, GRM6: metabotropic glutamate receptor, GRIA4: inotropic glutamate receptor AMPA type subunit GluR4, SNPs: single nucleotide polymorphisms, COMT: Catechol-O-methyltransferase, Val158Met: valine to methionine substitution at codon 158 [1-2,7,33-41]

Neurotransmitter type	Genes	Symptoms related and association
Serotonergic	5-HT2A receptor gene (T102C polymorphism)	Anxiety and depression
Serotonergic	5-HTT gene SLC6A4 (rs25531 + 5-HTTLPR)	Endogenous pain, cognitive and depressive symptoms, fatigue, and sleep disturbances
Dopaminergic	DRD4	Susceptibility to fibromyalgia
Dopaminergic	DAT1 polymorphism	Associated with sleepiness
GABAergic	GABRB3	Susceptibility to fibromyalgia and pain
Glutaminergic	Glutamine transporters gene (SLC1A5 and SLC25A22)	Upregulated in fibromyalgia
Glutaminergic	GRM6	Pain signaling
Glutaminergic	GRIA4	Central sensitization
Catecholaminergic	COMT (A-C-C-G haplotype) SNPs (rs6269, rs4633, rs4818, and rs4680)	Susceptibility to fibromyalgia and high pain sensitivity
Catecholaminergic	COMT Val158Met (rs4680) polymorphism	Anxiety, sleep disturbances, cognitive, and depressive symptoms, pain, and susceptibility to fibromyalgia.

**Table 2:** Immune/Inflammation-Associated Genes TNFα: tumor necrosis factor alpha, rs: reference single nucleotide polymorphism, IL: interleukin [7,35,39]

Genes	Symptoms related and association
TNFα rs1799964 polymorphism	Anxiety, depressive symptoms, and pain
TNFα rs1800629 polymorphism	Cognitive symptoms, fatigue, pain, and sleep disturbances
IL-6 rs1800795 polymorphism	Fatigue, pain, anxiety, and depressive symptoms
IL10, IL-25, 1L36A cytokine genes	Upregulated in fibromyalgia and in inflammatory processes

**Table 3:** Other Candidate Genes Associated to Fibromyalgia BDNF: brain-derived neurotrophic factor, rs: reference single nucleotide polymorphism, Val66Met: valine to methionine substitution at codon 66, SNP: single nucleotide polymorphism, SCN9A: voltage-gated sodium channel Nav1.7, TAAR1: trace amine-associated receptor 1, TSPO: translocator protein, TRPV2: transient receptor potential vanilloid 2, MTHFR: methylenetetrahydrofolate reductase, ACE: angiotensin-converting enzyme, I/D: insertion/deletion, MYT1L: myelin transcription factor 1-like, NRXN3: neurexin 3 [2,7,33-36,38-39,42]

Genes	Symptoms related and association
TT genotype of ​BDNF​ rs12273539	Susceptibility to fibromyalgia and enhance hyperalgesia
BDNF​ ​rs6265 (Val66Met SNP)	Fatigue, pain, sleep disturbances, anxiety, depressive, and cognitive symptoms
Sodium channel NaV1.7 (SCN9A)	Susceptibility to fibromyalgia and pain sensitivity
TAAR1 (rs8192619 SNP)	Pain sensitivity
TSPO (rs6971 SNP)	Pain and symptom severity
TRPV2	Susceptibility to fibromyalgia and impaired pain threshold
MTHFR (C677T polymorphism)	Stiffness and xerophthalmia
ACE I/D polymorphisms	Increased susceptibility to fibromyalgia
MYT1L	Cognitive symptoms and susceptibility to fibromyalgo
NRXN3	Susceptibility to fibromyalgia

More studies required to be done regarding genetic variants as the possible primary cause of fibromyalgia. We must know with exact certainty that we are actually dealing with a genetic disorder affecting both the immune system and the nervous system and perhaps reclassifying them.

Study limitation

Our article depends on reviewing free full-text research articles from the last 10 years, therefore there is a chance we have left out important information from paid full-text as well as from research articles published before 2010. We did not implement quality assessment of the chosen research studies. A systematic review was not performed.

## Conclusions

Currently, the etiology of fibromyalgia remains unknown. It is a challenging condition. This is in part due to the lack of a reliable and accurate diagnostic tools. Also, there are no specific biomarkers successfully linked to the root of this disease. The clinical profile of fibromyalgia overlaps with many psychiatric and rheumatology conditions, making it difficult to discard other causes of chronic pain before making a precise diagnosis. Furthermore, fibromyalgia can also present concomitantly with other pathologies associated with pain. With the help of new technology, scientists are engaged in multiple studies regarding fibromyalgia, with the hope of finding the specific precipitating factor that leads to its development. Future research should focus on the implication of pro-inflammatory cytokines in the pathophysiology of fibromyalgia and on the possible underlying influence of pain-related genes. In order to develop a more targeted therapy, to ease most of the symptoms, and to improve the daily activities affected in patients with fibromyalgia we need to know the exact mechanisms behind the pathophysiology of fibromyalgia.
